# Cerebral Aspergillosis in a Patient on Ibrutinib: A Case of Diagnostic Ambiguity and Multidisciplinary Care

**DOI:** 10.7759/cureus.115796

**Published:** 2026-09-04

**Authors:** Konstantinos Iason Tompler, Michael Osthoff

**Affiliations:** 1 Gastroenterology, University Hospital of Bern, Bern, CHE; 2 Department of Internal Medicine, Kantonsspital Winterthur, Winterthur, CHE

**Keywords:** aspergillus fumigatus, btk inhibitors, central nervous system infection, cerebral aspergillosis, ibrutinib, immunocompromised patients, mri brain lesions, voriconazole therapy

## Abstract

This case report describes the challenging diagnosis and management of cerebral aspergillosis in a patient with chronic lymphocytic leukaemia undergoing treatment with a BTK inhibitor. The initial presentation was deceptively unspecific, comprising cognitive changes, receptive aphasia, gait instability and mild neurological deficits without fever or significant systemic inflammation. The combination of a muted and non-indicative clinical picture and a broad differential diagnosis reflects the evolving complexity of CNS infections in patients treated with targeted haematological therapies.

Neuroimaging played a central role in directing attention toward an intracranial pathological process but did not resolve the ambiguity. CT suggested a space-occupying lesion, while MRI revealed multiple enhancing foci with mixed signal characteristics that could represent fungal, bacterial or neoplastic processes.

Diagnosis was only established after neurosurgical intervention, when resection of the dominant lesion provided tissue for definitive identification of *Aspergillus fumigatus*. Once the diagnosis was confirmed, long-term voriconazole therapy was initiated and adjusted with therapeutic drug monitoring, while ibrutinib was discontinued to prevent interactions and further immunosuppression. The patient’s clinical trajectory gradually stabilised, with radiological follow-up showing resolution of lesions and no recurrence.

Overall, this case illustrates the importance of maintaining a high index of suspicion for fungal CNS infections in patients receiving modern immunomodulatory treatments. The interplay between subtle and non-specific neurological clinical signs, atypical imaging and altered immune responses can delay diagnosis unless the clinical team actively integrates therapeutic background and invasive modalities into its reasoning. The favourable outcome achieved here underscores the value of early multidisciplinary involvement, timely pursuit of tissue diagnosis and careful coordination of antifungal therapy.

## Introduction

Aspergillus is a ubiquitous ascomycete that can cause a variety of clinical presentations depending on immune status. Invasive aspergillosis is well documented as the most common mold infection [1]. Central nervous system aspergillosis can be a fatal disease if detected late, which is often the case, as it goes along with non-specific clinical features [1]. Its clinical presentation can be subtle at first and then rapidly progressive. Even in severely immunocompromised patients, symptoms often mimic far more common neurological conditions, which delays the recognition of the underlying fungal aetiology. 

A recurring theme in the recent literature is the way CNS aspergillosis slips through conventional diagnostic frameworks. MRI patterns may hint at a fungal disease, but they rarely offer sufficient specificity for a firm diagnosis, especially when focal lesions overlap radiologically with pyogenic abscesses, metastases, or demyelinating processes [2,3]. Previous reviews had already highlighted the inherently heterogeneous appearance of granulomatous forms, particularly in immunocompetent hosts, which adds another layer of diagnostic ambiguity [4]. What has become increasingly clear, however, is that early suspicion - before the disease reaches an extensive stage - is crucial for a favourable outcome.

The emergence of Bruton tyrosine kinase (BTK) inhibitors such as ibrutinib has added an entirely new dimension to this problem. Ibrutinib is an oral BTK inhibitor used in chronic lymphocytic leukaemia and other B-cell malignancies. Although transformative in the management of chronic lymphocytic leukaemia, these agents disrupt innate antifungal immunity by inhibiting BTK-dependent innate immune responses, thereby increasing vulnerability to invasive Aspergillus species even without the traditional predisposing factors such as neutropenia or high-dose corticosteroids [5-7].

In neutrophils, BTK inhibition impairs fungal-induced NADPH oxidase activation, oxidative burst and phagocytosis of Aspergillus hyphae. In macrophages, inhibition of BTK interferes with NF-κB/NFAT-dependent inflammatory signaling and disrupts neutrophil recruitment [8]. 

In a large U.S. real-world study, 3.0% (38/1,286) of patients starting ibrutinib developed an invasive fungal infection within one year [9]. Case reports continue to accumulate, describing strikingly disseminated infections or unusually aggressive CNS involvement in patients who otherwise appeared clinically stable only weeks earlier [10,11]. Against this background, individual case reports remain essential for refining the clinical understanding of how cerebral aspergillosis presents, behaves and responds to therapy in the era of targeted haematological treatments [12-16].

This case illustrates a unique presentation of cerebral aspergillosis, initially characterized by nonspecific neurological deterioration in an immunocompromised patient. It underscores the importance of maintaining a high index of suspicion and pursuing brain tissue biopsy when clinically indicated, while recognizing the limited sensitivity and specificity of MRI findings.

## Case presentation

The present case report was prepared following the core principles of the CARE guidelines, with an emphasis on transparent, chronological and clinically meaningful presentation of the patient’s course after a written informed consent was obtained [17]. All clinical data were obtained directly from the patient’s electronic medical record, including the neurological examination, laboratory findings, radiological studies, microbiological analyses and operative reports. Rather than restructuring events into abstract categories, we preserved the natural sequence in which the symptoms emerged, investigations were pursued, and decisions were made. This approach allows the diagnostic reasoning to be seen as it occurred in real time, reflecting the uncertainty and evolving hypotheses that clinicians faced as the patient’s condition progressed.

No experimental interventions were performed. Identifying information was removed to preserve patient confidentiality, and the case was selected for publication because it illustrates diagnostic challenges that continue to emerge in patients treated with targeted haematological therapies.

Case study analysis: results

A 68-year-old, non-smoking, neurologically and cognitively healthy male known for a preexisting CLL since September 2015 and treated with ibrutinib 420 mg p.o once daily since January 2022 was referred by his general practitioner to the emergency ward at June 18, 2023 for further evaluation of a subacute, new neurological and mental state deterioration consisting of the simultaneous appearance of a sudden personality change, attention deficits, psychomotoric retardation, speech difficulties with receptor aphasia and worsening ataxia persistent for approximately three days between June 14 and 16 2023 with fluctuating severity. Clinical examination revealed scattered deficits, including impaired attention, episodic sensory aphasia of Wernicke-type, slurred articulation and a left-sided limb ataxia, suggesting an encephalopathic presentation rather than a stroke. The environmental and occupational exposure history was unremarkable.

Laboratory results (Table 1) did not show significant alterations; the neutrophilic granulocytes were mildly elevated (10.8 G/l, normal range 1.4-8.0 G/l) in the context of known lymphocytic leukocytosis (23.8 G/l, normal range 3.9-9.6 G/l). The initial CRP value was within normal limits (2 mg/l, normal range <5 mg/l).

**Table 1:** Laboratory results

Parameter	Result	Reference range
Leukocytes (WBC)	23.81 G/L	4.0-10.0 G/L
Neutrophilic granulocytes	10.8 G/L	1.4-8.0 G/L
Lymphocytes	11.31 G/L	1.07-3.12 G/L
Monocytes	1.0 G/L	0.16-0.95 G/L
Eosinophils	0.29 G/L	<0.7 G/L
Basophils	0.14 G/L	<0.15 G/L
Erythrocytes (RBC)	5.2 T/L	Men: 4.5-5.9 T/L
Hemoglobin	153 g/L	Men: 135-165 g/L
Hematocrit	44%	Men: 40-52%
Platelets (Thrombocytes)	183 G/L	150-400 G/L
CRP	2 mg/L	<5 mg/L
Creatinine	78 µmol/L	60-110 µmol/L (≈0.7-1.2 mg/dL)
Potassium (Kalium)	3.4 mmol/L	3.5-5.1 mmol/L
Sodium (Natrium)	134 mmol/L	136-145 mmol/L

At this stage, neither the clinical nor the laboratory profile pointed convincingly toward a fungal aetiology, but they also failed to support a straightforward cerebrovascular or bacterial explanation.

The non-contrast CT scan revealed a circumscribed hypodense lesion with indistinct margins, in the right frontopolar region, involving the gyrus rectus, olfactory structures, and orbital gyri, exhibiting an expansive character and causing slight deviation of the rostral midline because of mass effect and parietal oedema as well as a second hypodense lesion located subcentrally on the left side.

The MRI demonstrated and confirmed two distinct contrast-enhancing lesions (Figures 1, 2), measuring approximately 15 mm and 5 mm, with extensive surrounding oedema, and further sequences unveiled multiple bilateral parieto-occipital foci with extensive adjacent edema and atypical signal patterns - central diffusion restriction, a thickened peripheral capsule and focal susceptibility artefacts suggestive of blood degradation products or paramagnetic elements.

**Figure 1:**
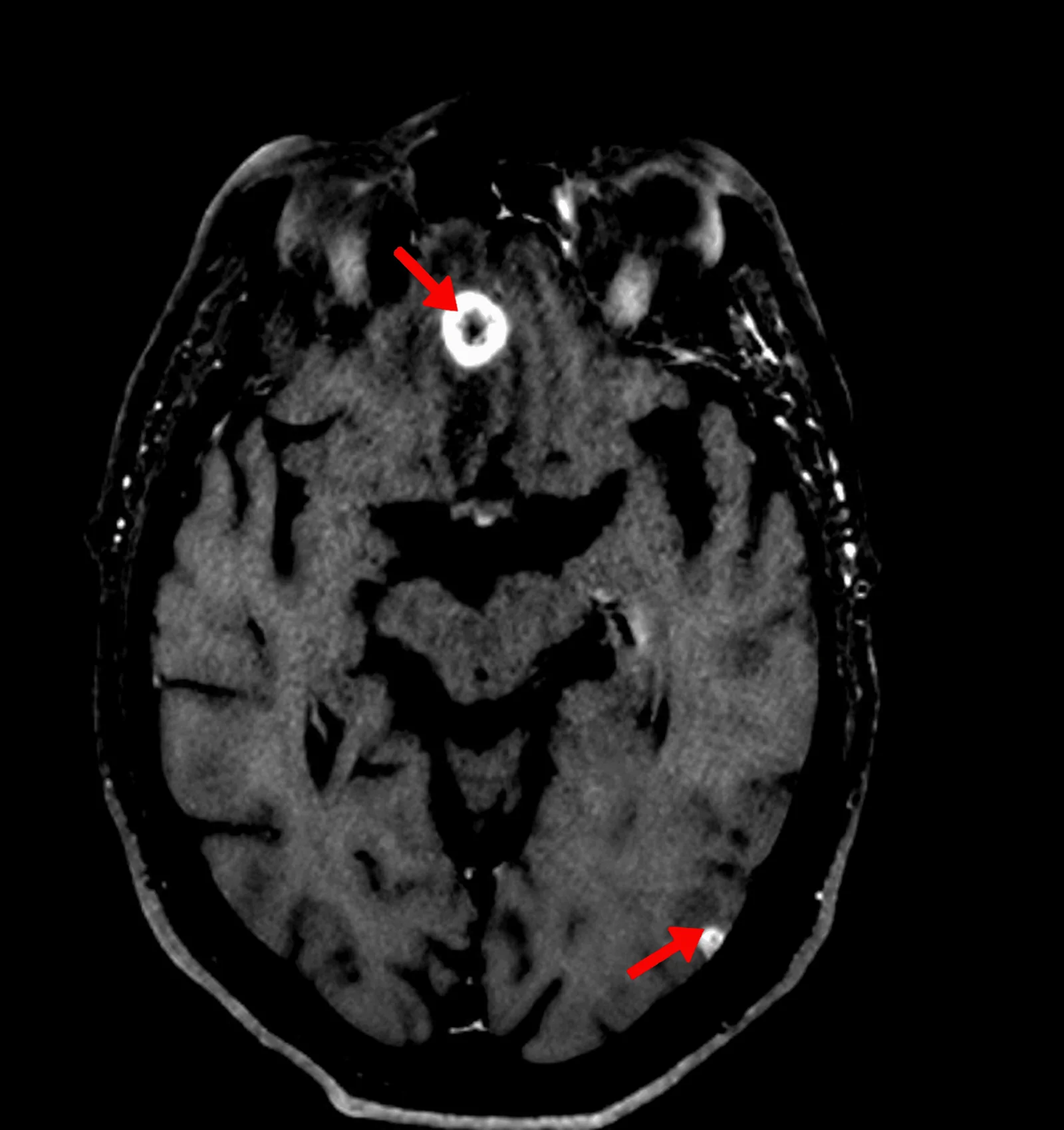
Axial T1-weighted image: ring-enhancing mass in the right frontal lobe and a smaller lesion in the left occipital lobe

**Figure 2:**
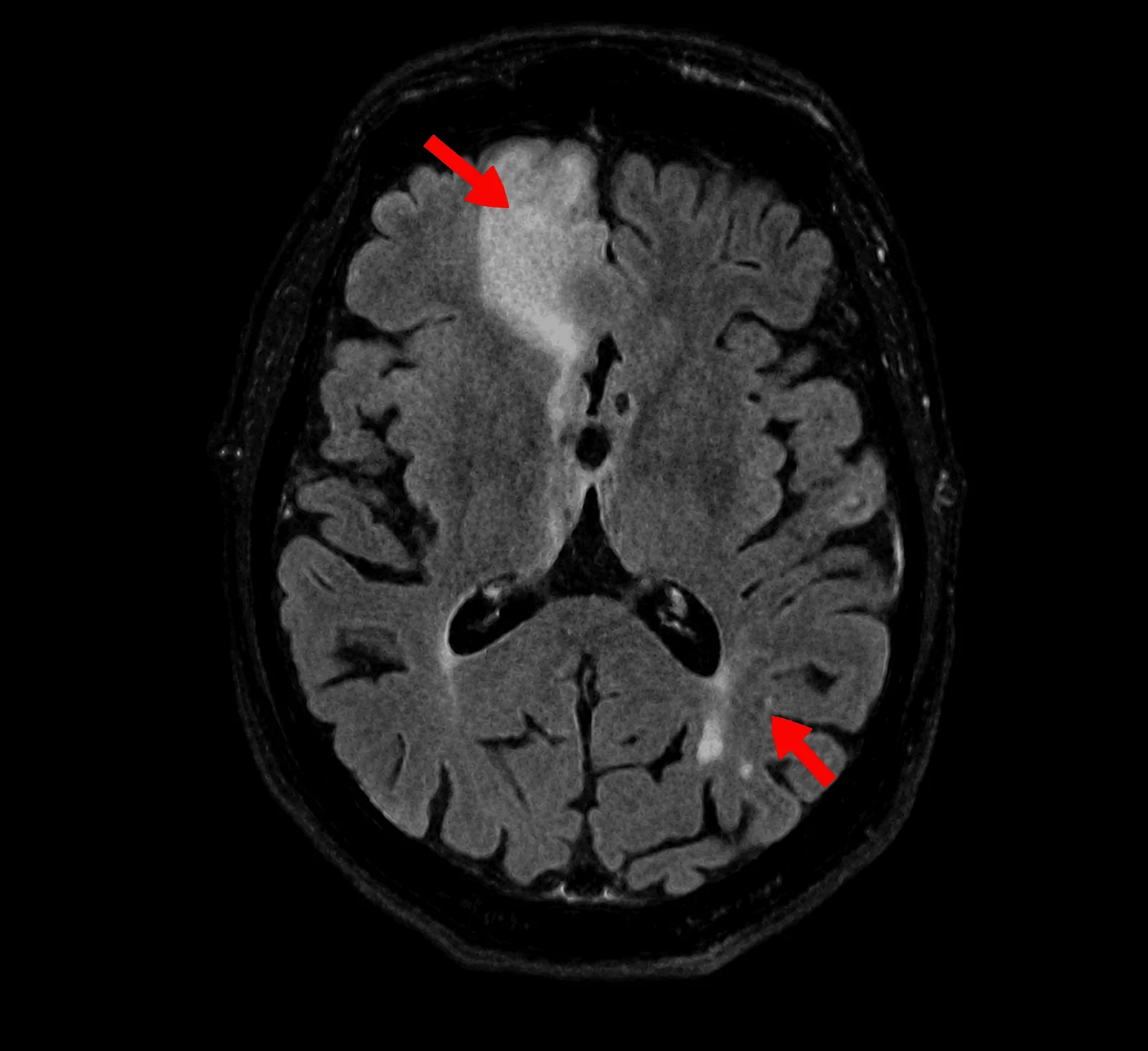
Axial FLAIR sequence; right frontal mass lesion with impressive tissue edema, smaller occipital lesion with adjacent edema

A PET-CT scan demonstrated multifocal metabolically active nodal supra- and infradiaphragmatic manifestations in the context of the CLL.

Instead of providing immediate diagnostic clarity, these findings widened the differential diagnosis considerably. The constellation could fit a manifestation of the known lymphoma, pyogenic abscesses, a manifestation of other metastatic disease, or fungal lesions, and the imaging pattern alone was insufficient to narrow the field.

The empirical antimicrobial therapy was therefore initiated considering an infectious etiology due to existing immunosuppression -ceftriaxone, metronidazole and voriconazole - paired with dexamethasone to address inflammatory oedema. Therapy with voriconazole was initiated with 200 mg twice daily. This therapeutic combination reflected the persistent uncertainty of the case: while bacterial meningitis remained a strong consideration, the possibility of a fungal abscess could no longer be ignored, especially given the patient’s treatment with ibrutinib, a drug known to subtly impair innate antifungal defences. 

The surgical intervention ultimately provided the definitive diagnostic breakthrough. A craniotomy and resection of the right frontobasal intracerebral space-occupying lesion was performed on June 28. This allowed, apart from the therapeutic effect, for histopathological examination (Figures 3, 4), which revealed granulomatous inflammation and necrotic tissue harbouring branching, septate fungal hyphae - features consistent with Aspergillus species. The subsequent culture confirmed Aspergillus fumigatus, transforming the clinical narrative from a broad differential to an unequivocal diagnosis of cerebral aspergillosis. With this confirmation, the antimicrobial regimen was narrowed to long-term monotherapy with voriconazole, adapted according to therapeutic drug monitoring to maintain plasma levels between 3.5 and 5.5 mg/L. According to the German national guidelines, the target trough level of voriconazole is approximately 2-6 mg/L (µg/mL) for cerebral aspergillosis [16].

**Figure 3:**
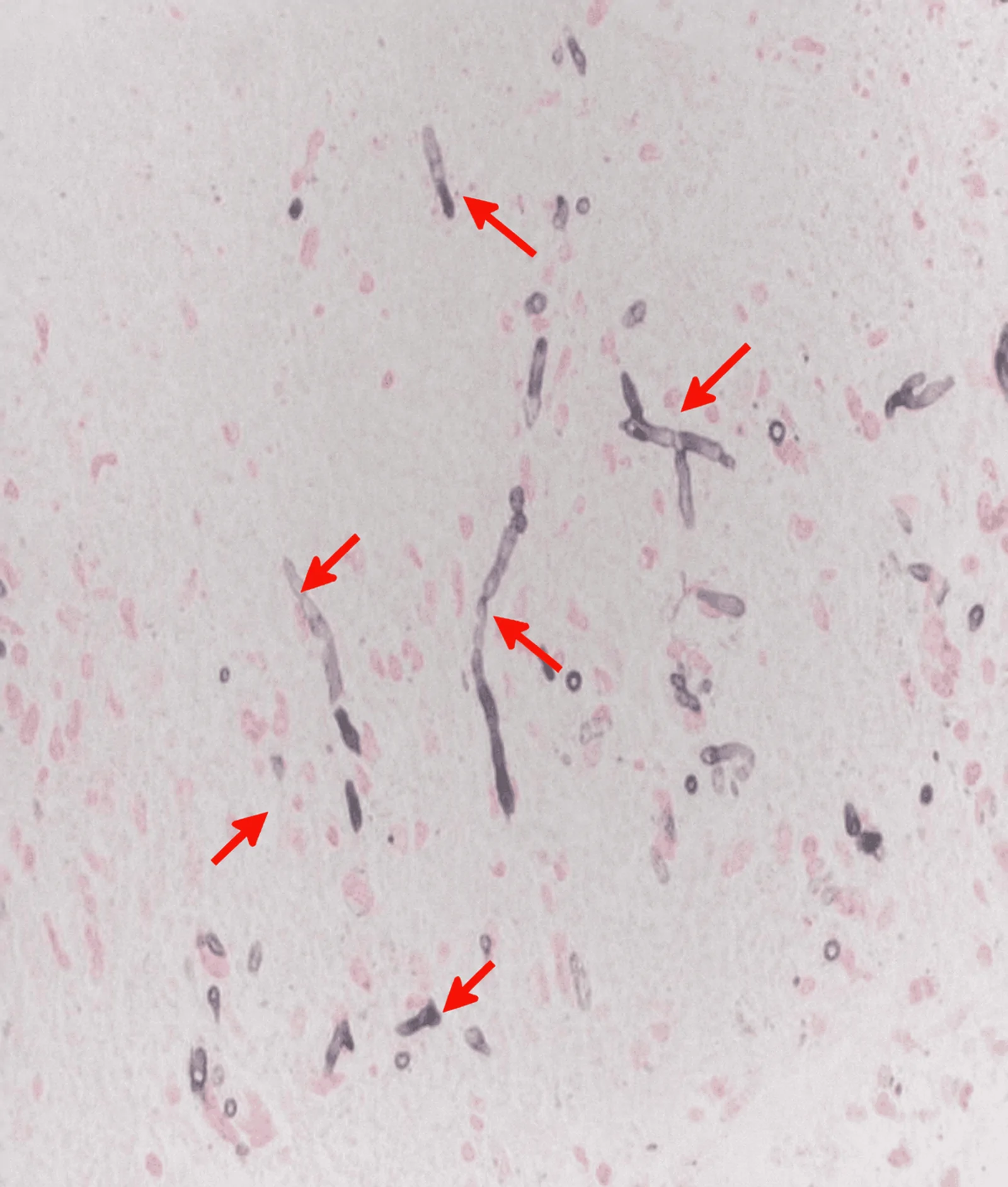
(x400) Brain tissue biopsy Grocott staining, branching, septate fungal hyphae

**Figure 4:**
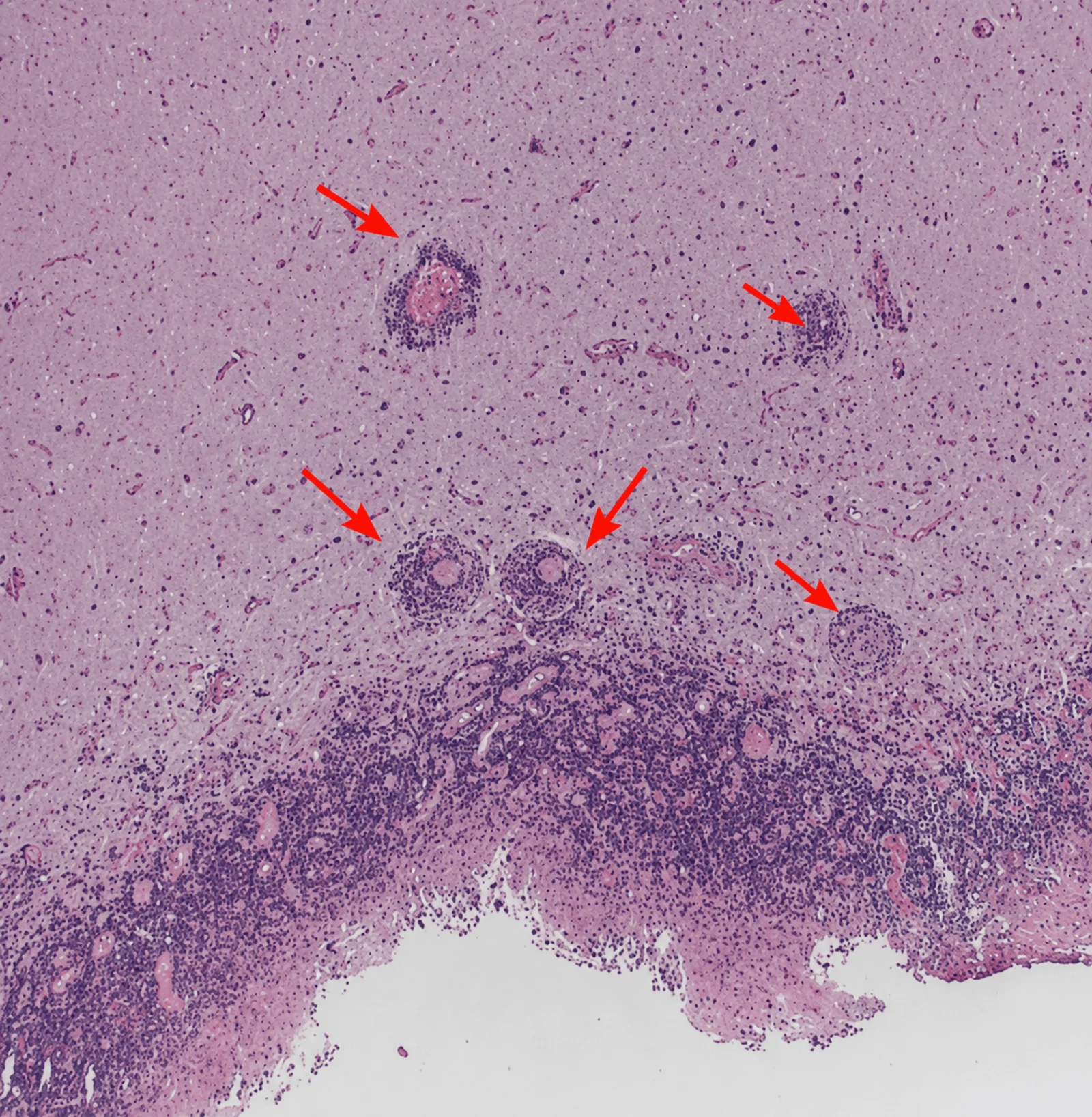
(x100) HE staining, cerebral tissue with lymphocytic and granulomatous inflammation

The voriconazole plasma concentration measured on July 4, 2023 was 5.1 mg/L.

A clinically relevant pharmacokinetic interaction exists between ibrutinib and voriconazole, as voriconazole is a strong CYP3A4 inhibitor and substantially increases ibrutinib exposure. Concomitant administration has been shown to increase maximum concentration of ibrutinib and area under the curve (AUC) by 6.7-fold and 5.7-fold, respectively, potentially increasing ibrutinib-related toxicity [17,18].

Ibrutinib was discontinued, not only because of the aforementioned interactions with azoles, but mainly because of its contribution to heightened fungal susceptibility, addressing a critical modifiable risk factor for treatment success. The therapeutic drug monitoring showed that the voriconazole levels were always within the target reference.

The clinical improvement unfolded gradually but consistently. Over the following weeks, the patient experienced a steady stabilisation of cognitive function and gait, despite a mild residual cognitive impairment that persisted. Radiological follow-up after five months demonstrated resolution of the enhancing lesions with no signs of recurrence, confirming both the adequacy of the surgical resection and the effectiveness of long-term antifungal therapy. During the six-month treatment with voriconazole, the patient reported some side effects consisting of visual disturbances, headaches, dizziness and nausea, pointing to a moderate tolerability. The clinical timeline is shown in Table 2.

**Table 2:** Chronological clinical timeline.

Time point	Clinical event/management/response
Day 0	Clinical manifestation
Day 3	Referral to the emergency department; CT scan performed
Day 3	Empirical therapy with ceftriaxone, metronidazole, voriconazole, and dexamethasone initiated
Day 3	MRI scan
Day 12	Neurosurgical operation
Day 13	Postoperative follow-up MRI
Day 15	Biopsy results and de-escalation to voriconazole monotherapy
Day 22	Discharge; transfer to a rehabilitation clinic
Day 31	Reduction of voriconazole dose to 200 mg twice daily
Day 180	Follow-up MRI showing resolution of the cerebral lesions

Due to the strong drug-drug interaction between ibrutinib and voriconazole, our hematology team considered the reintroduction of ibrutinib during voriconazole therapy to be associated with an unacceptably high risk of toxicity. Therefore, ibrutinib was not restarted during antifungal treatment.

Given the high risk of recurrent cerebral aspergillosis, secondary antifungal prophylaxis was considered essential following the completion of a six-month course of voriconazole therapy. This was particularly important because effective treatment of the underlying CLL made the reintroduction of ibrutinib therapy necessary, thereby potentially increasing the risk of recurrent cerebral aspergillosis.

For secondary prophylaxis, isavuconazole was initiated according to the following regimen: 200 mg three times daily for the first 48 hours (loading phase), followed by 200 mg once daily. Isavuconazole was selected because it has fewer clinically relevant drug-drug interactions with ibrutinib, thereby facilitating its subsequent resumption. Under secondary prophylaxis with isavuconazole, treatment with ibrutinib for the underlying B-cell lymphoma could be resumed at the standard dose. 

## Discussion

The patient’s initial presentation created a diagnostic landscape that was far less straightforward than expected. Although he arrived with clear functional deterioration - changes in personality, speech, cognitive impairment and an unsteady gait - the absence of fever, of indicative laboratory abnormalities and the relatively stable vital signs made the clinical picture deceptively calm. The turning point in the early evaluation emerged with neuroimaging, which showed a structural CNS pathology that did not pass to a cerebrovascular event and was suggestive of an infectious etiology. 

These contradictory elements highlight how radiological and biochemical findings in immunocompromised patients can deviate from textbook diagnostic patterns, forcing clinicians to revisit initial assumptions as new data emerge. The diagnostic step of the neurosurgical biopsy proved crucial, given the overlap of imaging characteristics with other intracranial entities and its insufficient specificity. This ambiguity underscored a central challenge in CNS aspergillosis: even advanced imaging can deliver more questions than answers, particularly when immunosuppression alters classic radiological features.

The clinical trajectory of this patient aligns with an increasingly recognised pattern of cerebral aspergillosis in individuals receiving targeted haematologic therapies. Several recent reports highlight how BTK inhibitors disrupt neutrophil and macrophage signalling, creating susceptibility to invasive Aspergillus spp. infections even in the absence of profound neutropenia or steroid exposure [9,19].

Our patient’s relatively mild systemic inflammatory response, despite extensive intracranial disease, mirrors this immunomodulated presentation. Similar cases described in CLL patients on ibrutinib also report initially muted symptoms that escalate abruptly, suggesting that immunological “silencing” rather than hyperinflammation characterises the early stages of infection in this group [6,7]. This reinforces the notion that clinicians should maintain a higher level of suspicion when neurological changes arise in patients treated with BTK inhibitors.

Neuroimaging provided important clues but did not yield a definitive diagnosis, a difficulty widely discussed in the literature. Fungal abscesses frequently mimic pyogenic lesions or neoplastic processes, and their radiological profile depends heavily on host immune status [3]. In our case, the combination of diffusion restriction, peripheral susceptibility artefacts and multilobar lesions created an ambiguous radiological picture - a pattern that resembles the heterogeneous MRI findings reported by Cavazza et al. [3] in immunosuppressed patients and by Ellenbogen et al. [4] in immunocompetent hosts with granulomatous disease. While some authors point to specific signal patterns as “suggestive” of fungal infection, the overlap across pathologies remains significant. The present case confirms that imaging alone rarely provides the level of certainty required for early and targeted antifungal treatment.

Collaboration among different disciplines is essential for both the diagnostic evaluation and the establishment of the diagnosis. Neurosurgery, radiology, infectious disease and pathology specialists all contributed materially to the management plan, each adding details that reshaped the diagnostic and therapeutic trajectory. Once clinicians have raised a suspicion of aspergillosis, careful analysis of imaging is crucial to accurately identify and interpret the radiological findings. However, the diagnosis can only be confirmed through histological examination of tissue samples obtained via neurosurgical intervention.

The diagnostic value of tissue sampling is also strongly supported by published evidence. Despite advances in fungal biomarkers and PCR assays, most studies continue to emphasise the central role of histopathology and culture in establishing a definitive diagnosis [4,5,12]. In our patient, it was only after surgical resection that the diagnosis of Aspergillus fumigatus was confirmed, echoing the approach described in other complex CNS cases where diagnosis was not confirmed without tissue [4,5,18]. This reliance on surgical confirmation has practical implications: although invasive, obtaining tissue may be the most efficient pathway to correct diagnosis when imaging features conflict with laboratory data or when empirical therapy must be chosen under uncertainty.

Therapeutic outcomes in this case were consistent with current recommendations advocating voriconazole as the agent of choice for CNS aspergillosis. Voriconazole remains a first option therapeutically because of its excellent CNS permeability and very good systemic absorption as well as because of the little resistance of Aspergillus spp and well-established therapeutic drug monitoring. The patient’s gradual improvement after initiation of antifungal therapy, coupled with radiological regression of the lesions and absence of recurrence at five months, reflects the response profile reported in a few contemporary case series [13,20]. Importantly, discontinuation of ibrutinib due to drug-drug interactions and bleeding risk reflects a key point raised both in mechanistic studies and clinical reports: BTK inhibition is not only a risk factor for infection but also complicates antifungal management through CYP-mediated interactions. Cases described by McCarter et al. [12] underline how continued ibrutinib during infection may worsen dissemination, a scenario avoided here through timely withdrawal of the agent.

It should be stressed that, when comparing this case to recent literature, a broader pattern becomes visible: CNS aspergillosis in immunocompromised patients is shifting from a rare diagnosis to one that clinicians increasingly encounter, often presenting with subtle neurological symptoms and atypical imaging. Multiple authors stress that earlier identification depends on integrating clues - modest inflammatory markers, ambiguous MRI patterns, unexpected neurological changes and the presence of modern immunomodulatory therapies - rather than waiting for overt signs of fulminant infection [1,5,6,18].

Finally, the trajectory of the case highlights the delicate balance between early diagnostic suspicion and the need for confirmatory tissue diagnosis in immunocompromised patients, especially those receiving targeted therapies. It also underscores the importance of multidisciplinary management; radiology, neurology, infectious disease specialists and neurosurgeons each played a decisive role in steering the patient toward recovery.

The outcome in our case, marked by early neurosurgical intervention and careful therapeutic monitoring, illustrates how rapid multidisciplinary coordination can turn a potentially fatal CNS opportunistic infection into a manageable clinical problem. At the same time, it highlights a continuing need for heightened awareness, particularly as targeted treatments reshape the infectious risk landscape of haematologic patients.

## Conclusions

Ibrutinib and other newer immunosuppressants can increase the risk of mycotic infections. Neurological symptoms in immunosuppressed patients have a broad differential diagnosis, including opportunistic infections. MRI can provide clues, but a biopsy is often necessary. Voriconazole is the treatment of choice for central nervous system aspergillosis and its correct administration regimen combined with surgical diagnosis/intervention and multidisciplinary management can lead to a favourable outcome. 
